# EmotionBox: A music-element-driven emotional music generation system based on music psychology

**DOI:** 10.3389/fpsyg.2022.841926

**Published:** 2022-08-29

**Authors:** Kaitong Zheng, Ruijie Meng, Chengshi Zheng, Xiaodong Li, Jinqiu Sang, Juanjuan Cai, Jie Wang, Xiao Wang

**Affiliations:** ^1^Key Laboratory of Noise and Vibration Research, Institute of Acoustics, Chinese Academy of Sciences, Beijing, China; ^2^University of Chinese Academy of Sciences, Beijing, China; ^3^State Key Laboratory of Media Convergence and Communication, Communication University of China, Beijing, China; ^4^School of Electronics and Communication Engineering, Guangzhou University, Guangzhou, China; ^5^School of Humanities and Management, Southwest Medical University, Luzhou, China

**Keywords:** emotional music generation, deep neural networks, auditory perceptions, music psychology, music element

## Abstract

With the development of deep neural networks, automatic music composition has made great progress. Although emotional music can evoke listeners' different auditory perceptions, only few research studies have focused on generating emotional music. This paper presents EmotionBox -a music-element-driven emotional music generator based on music psychology that is capable of composing music given a specific emotion, while this model does not require a music dataset labeled with emotions as previous methods. In this work, pitch histogram and note density are extracted as features that represent mode and tempo, respectively, to control music emotions. The specific emotions are mapped from these features through Russell's psychology model. The subjective listening tests show that the Emotionbox has a competitive performance in generating different emotional music and significantly better performance in generating music with low arousal emotions, especially peaceful emotion, compared with the emotion-label-based method.

## Introduction

Computational modeling of polyphonic music has been deeply studied for decades (Westergaard et al., 1959). Recently, with the development of deep learning, neural network systems for automatic music generation have made great progress on the quality and coherence of music (Herremans et al., 2017; Herremans and Chew, 2019; Jin et al., 2020). As we know, emotion is of great importance in music since the music consistently elicits auditory responses from its listeners (Raynor and Meyer, 1958). Therefore, Emotional music has significant implications for subjects such as music psychology, music composition, and performance. However, surprisingly, automatic systems rarely consider emotion when generating music, which lacks the ability to generate music that evokes a specific auditory response.

To study the automatic music generation with music psychology, it is necessary to review the relation between music emotions and music elements. As mentioned by Parncutt (2014), the relationship in Western tonal music between emotional valence (positive vs. negative) and music-structural factors, such as tempo (fast vs. slow) and mode (major vs. minor tonality), have been studied. Experimental results have illustrated that a fast tempo tends to make music sound happy while slow tempo has the opposite effect (Rigg, 1940). In typical tonal musical excerpts, the experimental result showed that tempo was more determinant than the mode in forming happy-sad judgments (Gagnon and Peretz, 2003). Many experiments have demonstrated that musical excerpts written in the major or minor mode were judged to be positive or negative, respectively (Hevner, 1935, 1936). Recent psychological studies have shown that the happiness ratings were elevated for fast-tempo and major-key stimuli while sadness ratings were elevated for slow tempo and minor-key stimuli (Hunter et al., 2008, 2010). Another study has revealed that mode and tempo were the most impactful cues in shaping emotions while sadness and joy were among the most accurately recognized emotions (Micallef Grimaud and Eerola, 2022). The effect of cues on emotions in music as combinations of multiple cues rather than as individual cues has also been discussed, as mixed cues might portray a complicated emotion.

Most previous emotional music generation models were based on emotion labels (Ferreira and Whitehead, 2019; Zhao et al., 2019; Ferreira et al., 2020), without taking into consideration the effect of music psychology. Moreover, label-based methods require a huge music dataset labeled with different emotions, which need a lot of tedious work. Utilizing music psychology instead of the manual labels to train the emotional music generator and exploring the most suitable music elements for evoking the specific emotion are the main focuses in this paper.

In this work, we extract two features from two music elements (i.e., tempo and mode) to supervise the deep neural network for generating music with a specific emotion. To the best of our knowledge, this is the first music-element-driven emotional symbolic music generation system based on a deep neural network.

## Related work

Currently, deep learning algorithms have become mainstream methods in the field of music generation research. Music generation can be classified into two types: symbol domain generation (i.e., generating MIDIs or piano sheets Yang et al., 2017; Dong et al., 2018) and audio domain generation (i.e., directly generating sound waves van den Oord et al., 2016; Schimbinschi et al., 2019; Subramani et al., 2020).

Recurrent Neural Network (RNN) or its variants have been widely used to model sequential data. Its outstanding temporal modeling ability makes it suitable for music generation. The first attempt is that Todd used RNN to generate monophonic melodies early in Todd (1989). To solve the gradient vanishing problem of RNN, Eck et al. proposed an LSTM-based model in music generation for the first time (Eck and Schmidhuber, 2002). In Boulanger-Lewandowski et al. (2012), RNN combined with Restricted Boltzmann Machines was proposed to model polyphonic music, which is superior to the traditional model in various datasets. In 2016, the magenta team proposed the Melody RNN model which can generate long-term structures in songs (Waite, 2016). In 2017, anticipate RNN (Hadjeres and Nielsen, 2017) was used to generate music interactively with positional constraints. Moreover, Bi-axial LSTM (BALSTM) (Johnson, 2017) proposed by Johnson et al. are capable of generating polyphonic music while preserving translation invariance of the dataset. Recently, more advanced deep generative models, such as VAE (Hadjeres and Nielsen, 2017; Brunner et al., 2018), GAN (Guan et al., 2019; Huang et al., 2019), and Transformer (Huang et al., 2019; Zhang, 2020), have gradually been used in music generation.

The expressive generation has long been explored in the field of computer music, reviewed in Kirke and Miranda (2009). With the development of deep learning, there are several previous attempts to generate emotional music based on deep neural networks. Ferreira et al. proposed a multiplicative long short-term memory (mLSTM) based model that can be directed to compose music with a specific emotion and analyze music emotions (Ferreira and Whitehead, 2019). mLSTM is a RNN architecture for sequence modeling that combines the factorized hidden-to-hidden transition of multiplicative RNN with the gating framework from the LSTM. However, only video game soundtracks are used in training and evaluation. In 2019, Zhao et al. extended the BALSTM network proposed in Mao (2018) and used the model in emotional music generation (Zhao et al., 2019). Recently, Ferreira et al. proposed a system called Bardo Composer, which generates music with different emotions for the tabletop role-playing games based on the mood of players (Ferreira et al., 2020). However, all methods mentioned above are label-based thus a large dataset labeled with emotions is needed. Moreover, to the best of our knowledge, no MIDI dataset labeled with emotion is available online. Labeling the dataset manually takes a lot of time and effort. In our work, we train the model on an open-source MIDI dataset without emotion labels.

## Data preprocessing

### Note representation

The input of our proposed generation model consists of polyphonic MIDI files, which are composed of both melody and accompaniment. To present notes with expressive timing and dynamics, we use the performance encoding proposed in Oore et al. (2020), which consists of a vocabulary of NOTE-ON, NOTE-OFF, TIME-SHIFT, and VELOCITY events. The main purpose of encoding is to transform the music information in MIDI files into a suitable presentation for training the neural network.

The pitch information in MIDI files ranges from 0 to 127, which is beyond the pitch range of a piano. In our work, pieces in the training set are all performed by piano. Thus, the pitch range is only presented from 21 to 108, which corresponds to A0 and C8 on piano, respectively. For each note, music dynamics is recorded in MIDI files, ranging from 0 to 127 to present how loud a note is. For convenience, we use velocity ranges from 0 to 32 to convey the dynamics. The range can be mapped from 0 to 127 when generating MIDI files.

Finally, a MIDI excerpt is represented as a sequence of events from the following vocabulary of 240 different events:

- 88 NOTE-ON events: one for each of the 88 (21-108) MIDI pitches. Each event starts a new note.
- 88 NOTE-OFF events: one for each of the 88 (21-108) MIDI pitches. Each event releases a note.
- 32 TIME-SHIFT events: each event moves the time step forward by increments of 15 ms up to 1 s.
- 32 VELOCITY events: each event changes the velocity applied to all upcoming notes.

### Feature extraction

In this work, the model is fed with two extracted musical features, namely pitch histogram and note density. All these calculations are done automatically by computers and thus no human labors are required. A pitch histogram (Tzanetakis et al., 2003) is an array of 12 integer values indexed by 12 semitones in a chromatic scale, showing the frequency of occurrence of each semitone in a music piece. An example of a pitch histogram in C major is shown in Table 1. According to music theory, notes with a sharp sign are not included in C major. Therefore, in this work, we set their corresponding value in pitch histogram as 0 so that they will never be played in a C major music. C, F, and G are the tonic, subdominant, and dominant in C major, respectively. They are the main elements in a C major music so their corresponding value in pitch histogram is set as 2, which means the probability of starting these notes is two times as much as other notes in C major. Pitch histograms can capture musical information regarding harmonic features of different scales.

**Table 1:** An example of a pitch histogram in a C major scale.

**Pitch name**	**C**	**C^♯^**	**D**	**D^♯^**	**E**	**F**	**F^♯^**	**G**	**G^♯^**	**A**	**A^♯^**	**B**
Pitch histogram	2	0	1	0	1	2	0	2	0	1	0	1
Probability distribution	0.2	0	0.1	0	0.1	0.2	0	0.2	0	0.1	0	0.1

♯ Means higher in pitch by one semitone.

Note density is a number to record how many notes will be played within a time window (2 s in our work). Note density can present the speed information in each part of a music piece. Note density and pitch histogram are calculated at each time step.

The motivation for this is that we can explicitly choose a pitch histogram and note density when creating samples, which provides us with two options to control the music generation. By changing the pitch histogram and note density, we can therefore alter the mode and tempo of the music, which ultimately leads to emotional difference.

### Russell emotion model

**Figure 1:**
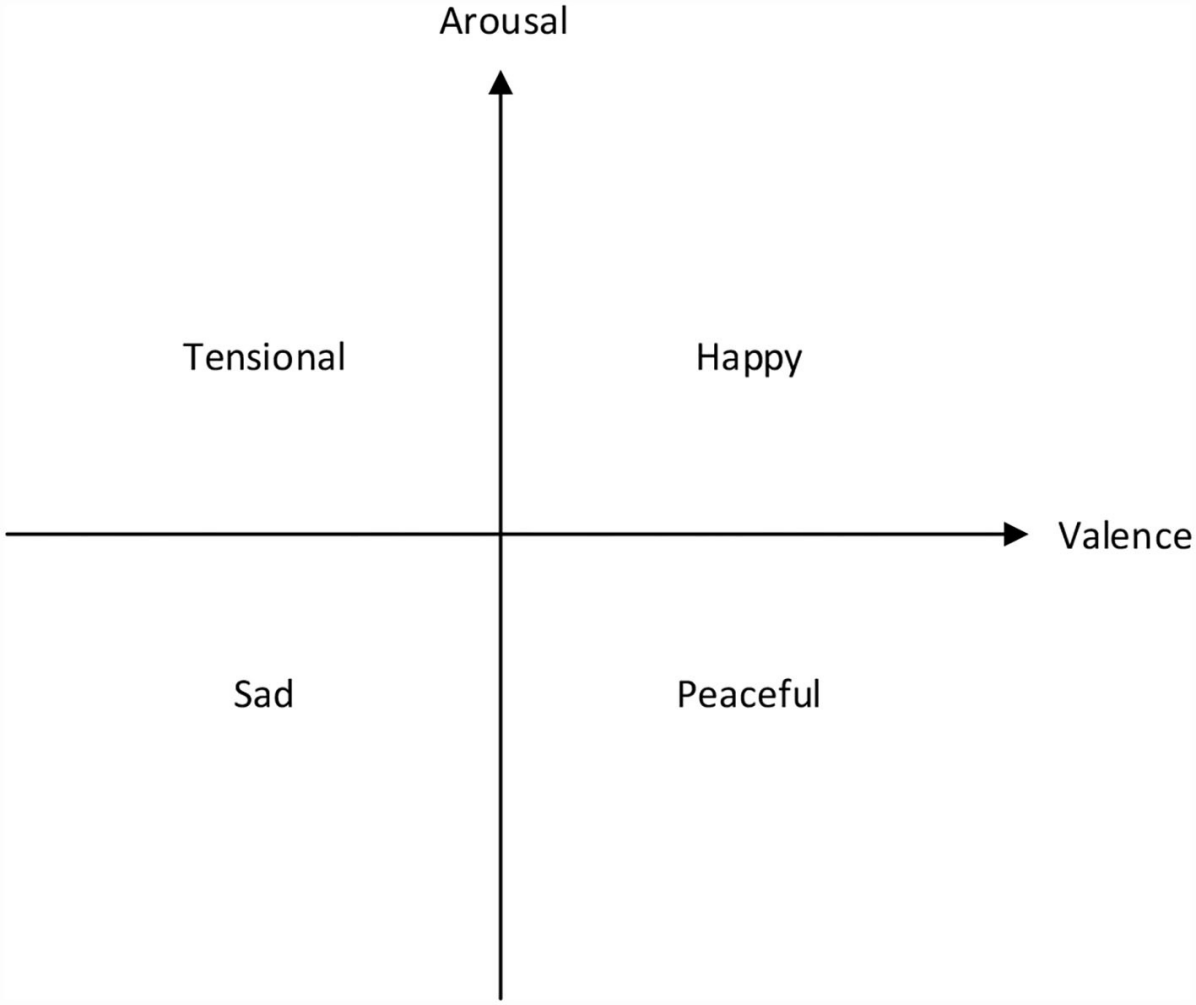
Simplified Russell's two-dimensional valence-arousal emotion space. The x-axis denotes valence while the y-axis denotes arousal.

There are various models for describing emotion and they can be mainly divided into four categories: discrete, dimensional, miscellaneous, and music-specific models (Eerola and Vuoskoski, 2012). This work is based on the simplified emotion model of Russell (1980). Russell's circumplex model is a typical dimensional model, which uses two coordinate axes to present the degree of valence and arousal, respectively. This emotion model is shown in Figure 1. For simplicity, we only use four basic emotions as shown in four quadrants. Our model is designed to generate music with these four basic emotions, namely happy, tensional, sad, and peaceful. The four emotions are located in four different quadrants, presenting four varying degrees of valence and arousal.

### Emotion presentation

As we have mentioned in the introduction, there is a strong connection between music elements and music emotional valence. Therefore, we combine note density and pitch histogram to control the tempo and mode of the generated sample. According to twelve-tone equal temperament, an octave is divided into 12 parts, all of which are equal on a logarithmic scale. So, we can choose the mode when generating music by changing the probability of each semitone. We use an array containing 12 integers to present a pitch histogram. For example, C major is presented as [2, 0, 1, 0, 1, 2, 0, 2, 0, 1, 0, 1] where 2 presents the tonic, subdominant, and dominant while 1 presents other notes in the scale. Pitch histogram of C minor is presented as [2, 0, 1, 1, 0, 2, 0, 2, 1, 0, 1, 0] according to music theory. A pitch histogram is used to control the valence of music.

Note density indicates the number of notes that will be performed within 2 s (the time window is adjustable). We set note density as 1 to present slow music and note density as 5 to present fast music. Note density is used to control the arousal of music. Combining mode and note density as two adjustable parameters, we aim to generate four categories of emotional music: happy (with the major scale and fast tempo), tensional (with the minor scale and fast tempo), peaceful (with the major scale and slow tempo), and sad (with the minor scale and slow tempo).

## Method

### Neural network architecture

**Figure 2:**
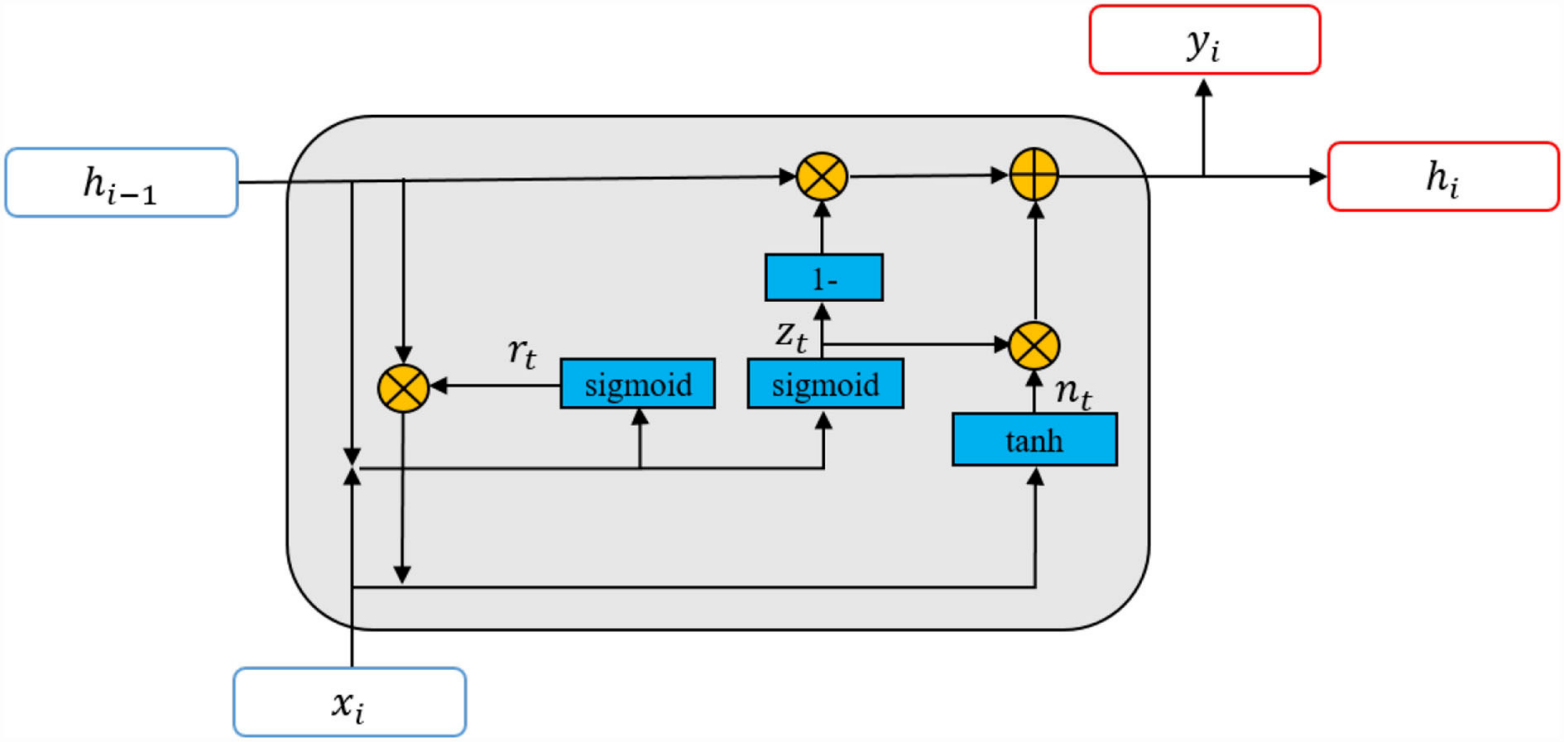
The illustration of gated recurrent units (GRU). *x*_*i*_ and *y*_*i*_ denote the current input and output of GRU, *h*_*i*−1_ and *h*_*i*_ are the last hidden information and current hidden information, *r*_*i*_ and *z*_*i*_ are the reset and update gates. A GRU network is formed from a series of GRUs.

A recurrent neural network has an excellent performance in modeling sequential data. A gated recurrent unit (GRU) (Cho et al., 2014) is an improved version of the standard RNN. It was proposed to solve the vanishing gradient problem of a standard recurrent neural network during backpropagation. The gating mechanism enables GRU to carry information from earlier time steps to later ones. The illustration of GRU is shown in Figure 2. In our work, GRU is used for temporal modeling.

**Figure 3:**
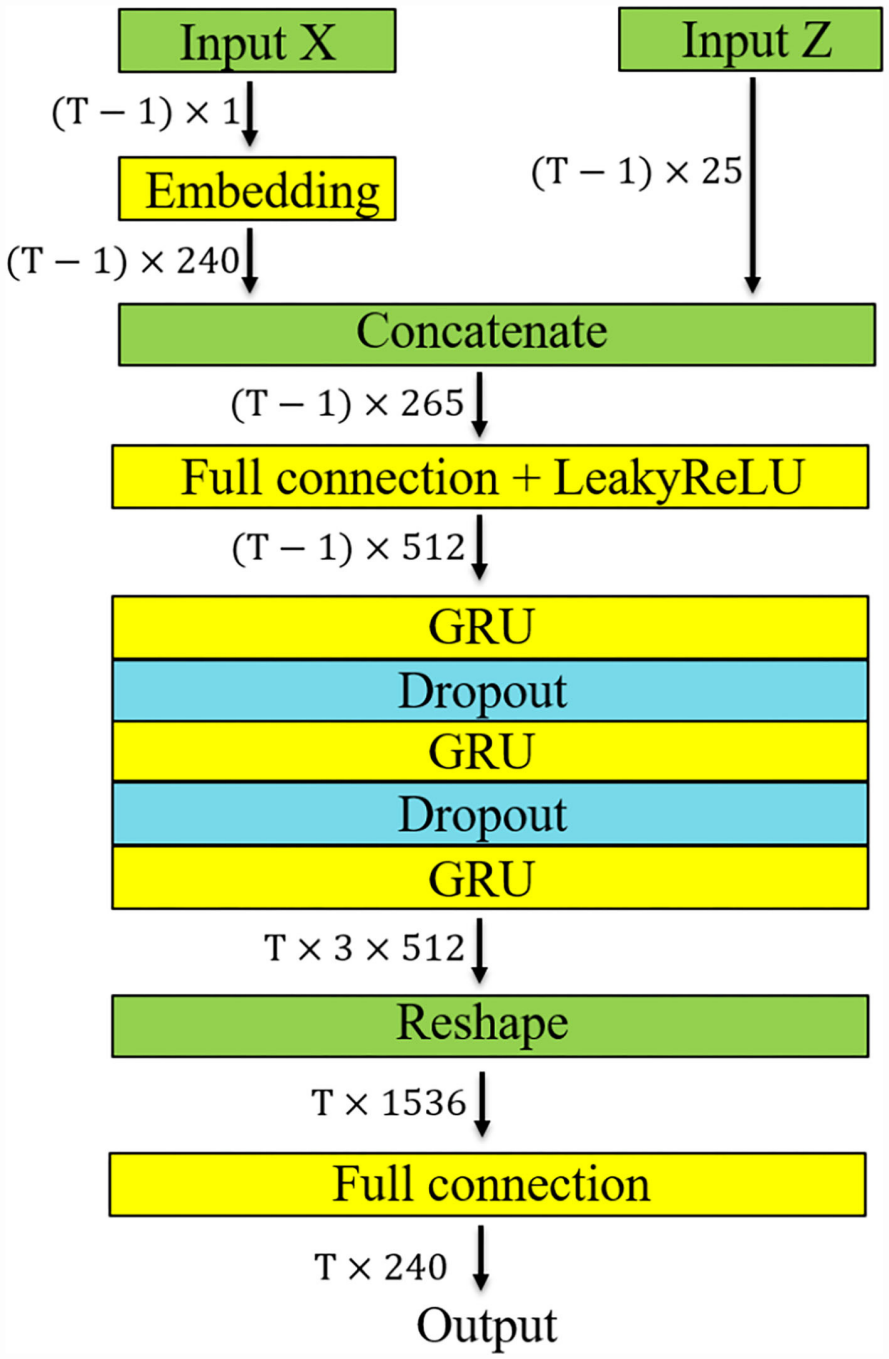
Diagram of the EmotionBox model architecture. “Input X” denotes a sequence of events and “Input Z” denotes the pitch histogram and note density.

The model is shown in Figure 3. Input X represents the masked performance events while Input Z represents the pitch histogram and the note density. Masking means the last event of each event sequence is dropped out and the rest part of the event sequence is sent to the neural network as the input. The reason for this is to make the model generate the unmasked sequence recursively. Then, we can calculate the loss, i.e., the difference, between the generated unmasked sequence and ground truth. If the length of an event sequence is *T*, the size of Input X (i.e., the masked performance events) will be (*T*−1) × 1. Each performance event is converted to a 240-dimension vector by a 240 × 240 embedding layer. The 240-dimension vector was chosen for convenience. The pitch histogram is a (*T*−1) × 12 vector and note density is converted to a (*T*−1) × 12 one-hot vector. A (*T*−1) × 1 zero vector is used to increase the stability of the neural network. Therefore, the size of input Z is (*T*−1) × 25.

The pitch histogram and note density are then concatenated with the 240-dimension vector. The size of the concatenated vector is (*T*−1) × 265. The concatenated input is fed into a 265 × 512 full connection layer and a rectified linear unit (ReLU) activation function. Then, this (*T*−1) × 512 vector is sent into a three-layer, 512-unit GRU, with a 0.3 dropout applied after each of the first two GRU layers. The GRU output is then fed to a 240-unit linear layer. The output of the neural network is a *T*× 240 vector. The output presents the probability of each event at each time step. The cross-entropy loss between the generated sequence and the unmasked event sequence, namely, the ground truth, is then calculated. The codes of this work have been open-sourced on Github^1^.

### Emotional music generation

At the generating stage, we generate samples with different emotions by specifying a particular pitch histogram and note density. When the model generates music, the first event will be randomly selected. The first event, pitch histogram, and note density are sent to the model to create new events recursively. The output of our model is the probability of 240 events. If we use greedy sampling to select an event with the largest probability, the sample may end up with some partial repetition, which means a small part of the music may repeat again and again. Therefore, we combine greedy sampling with stochastic sampling. We select a threshold ranged from 0 to 1. Whenever a new event is sampled, we produce a random number ranged from 0 to 1. If the random number is larger than the threshold, this event will be sampled using the greedy algorithm, which means selecting an event with the largest probability. If not, this event will be sampled based on the probability of each event, which produces a lot of uncertainty.

When generating a new piece of emotional music, we can use temperature (He et al., 2018) to alter the degree of uncertainty. Temperature is a hyperparameter used to control the randomness of predictions by scaling the logits before applying softmax. Lower temperature results in more predictable events, while higher temperature results in more surprising events. The temperature parameter is manually tuned by listening to the generated music. If the music is too random, the temperature will be turned down. If the music is too repetitive, the temperature will be turned up.

## Experiment

### Dataset

We selected a widely used dataset, piano-midi^2^, to train our model. It includes 329 piano pieces from 23 classical composers. Each piece is a MIDI file capturing a classical piano performance with expressive dynamics and timing. The dataset is highly homogeneous because all of the pieces in it are classical music, and the solo instrument is consistently piano. The authors in Zhao et al. (2019) labeled this dataset with four basic emotions mentioned above (i.e., happy, tensional, peaceful, and sad) manually to train their label-based automatic emotional music generator. For the comparison experiment, we also used this emotion-labeled dataset with the permission of the authors to train a label-based model. The Pretty-Midi package was used to extract the note information from the MIDI files (Raffel and Ellis, 2014).

### Training

At the training stage, the whole sequence of events is cut into 200-event-wide event sequences. The stride of event sequences is 10 events. The network was trained using the ADAM optimizer with a loss function of cross-entropy loss between the predicted event and the ground truth event. We used a learning rate of 0.0002, and the model was trained for 100 epochs with a batch size of 64. We implemented our models in PyTorch.

### Comparison

We implement a label-based model for comparison as all previous emotional music generation models were based on emotion labels (Ferreira and Whitehead, 2019; Zhao et al., 2019). In order to evaluate the performance between our proposed method and the labeled-based method, the structure of the label-based model remains unchanged except that the inputs Z of the model are substituted with emotion labels. One-hot coding is used to present four basic emotions. The neural network is trained to learn the mapping between music emotions and well-classified emotion labels. In the generation stage, the label-based model takes the emotion label as input.

## Results and discussion

To evaluate the performance of music generation given a specific emotion, a subjective listening test study was carried out to compare our proposed method with the label-based method. Similar to the subjective listening test for analyzing different styles of classification, three 6-s long music samples were provided for each emotion and each model^3^. The total amount of music samples was 24 (3 samples × 4 emotions × 2 models). The samples were randomly selected and shuffled. Table 2 shows the average note density of the experimental stimuli. Twenty-six subjects took part in the test. For each sample, participants were asked which emotion was observed in the sample? They have to choose one option from happy, peaceful, sad, and tensional. It is a little difficult for untrained participants to classify the music's emotion. Therefore, we provided a warming-up stage by playing four manually selected emotional music samples with their corresponding emotional labels. During the listening test, samples can be stopped and replayed to make sure the participants can hear the music clearly.

**Table 2:** The average note density of the experimental stimuli.

	**EmotionBox**	**Label-based method**
Happy	18.03	20.54
Tensional	17.23	32.39
Sad	6.24	12.06
Peaceful	6.29	14.41

### Emotion classification

**Figure 4:**
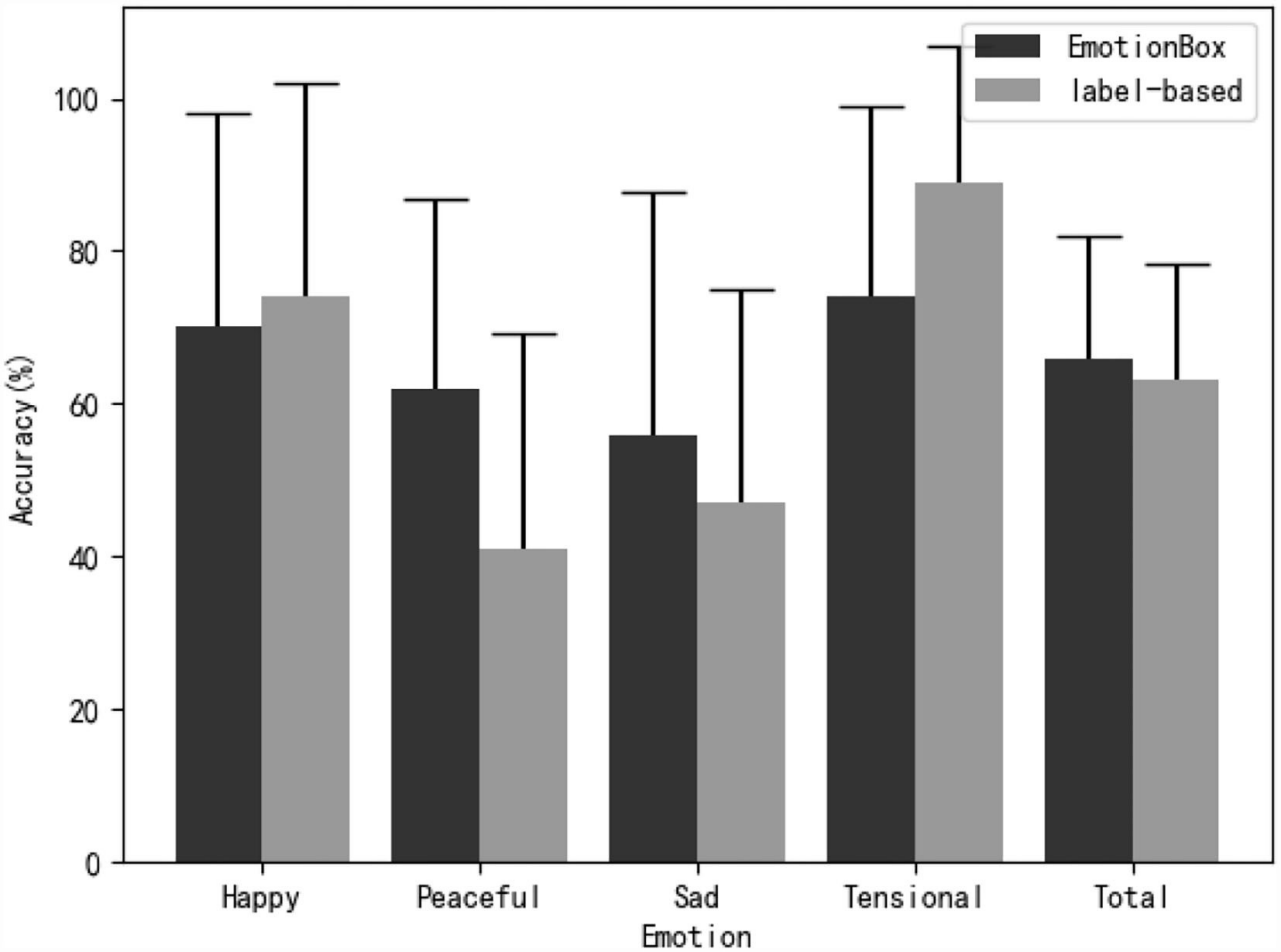
The mean accuracy and SD of subjective evaluation test for classifying generated music samples into emotion categories.

In this section, we calculated the accuracy of emotion classification for each of the four emotions and two methods. The statistical results are shown in Figure 4. In Figure 4, it shows that our proposed model, without a database labeled with emotions, has comparable performance to the label-based model in terms of emotion classification accuracy. Among the four kinds of emotion, the results indicate that the music samples with tensional and happy emotions were correctly recognized by the highest accuracy for both methods. These observations can be explained by an emotion psychology study that showed that valence can be distinguished more easily by high-arousal stimuli (Bradley et al., 2001). The proposed method outperforms the label-based method on peaceful and sad samples, which greatly overcome the shortcomings of the label-based method and yield a more balanced result. A two-way ANOVA is used with emotion (happy, sad, tensional, peaceful) and model (EmotionBox, label-based) set as within-subject factors to investigate how these two factors, in combination, affect the accuracy of subjective experiments. For each subject, the accuracy of emotion classification was calculated for each emotion and model. The classification accuracy was calculated by dividing the number of samples that were correctly recognized by the number of samples tested for each emotion and model (3 tested samples for each emotion and model). The statistical results show that model [*F*_(1, 25)_ = 0.603, *p* = 0.445, partial η^2^ = 0.024] has no significant effect while emotion [*F*_(3, 75)_ = 15.115, *p* < 0.01, partial η^2^ = 0.377] has a significant effect on the accuracy of subjective experiments. For the interaction of model and emotion, Mauchlys test of sphericity indicates that the assumption of sphericity has been violated [$\chi_{\left(5\right)}^{2}=12.904,p=0.024$]. By applying the Greenhouse-Geisser correction, the interaction of model and emotion shows a significant effect on the accuracy of subjective experiments [*F*_(2.435, 60.865)_ = 6.475, *p* < 0.01, partial η^2^ = 0.206].

Table 3 shows a ***post-hoc*** Bonferroni adjusted pairwise comparison within each emotion pair of two methods. Table 3 indicates that there are significant differences between the two methods on tensional and peaceful samples. The emotion classification accuracy of the label-based method is significantly high on tensional emotion while that is significantly low on peaceful emotion. There are no significant differences between the two methods on happy and sad samples. The note density of experimental stimuli can be used to explain why the proposed model achieved good performance for peaceful whereas the label-based model worked well for tensional. Table 2 shows that the tensional samples of the label-based model have a much higher note density than that of the EmotionBox. Therefore, the subjects are more likely to judge the former as tensional. On the other hand, the peaceful samples of the EmotionBox have a much lower note density than that of the label-based model. Therefore, the subjects are more likely to judge the former as peaceful. A ***post-hoc*** Bonferroni adjusted pairwise comparison between each emotion of EmotionBox has been conducted. The result shows no statistically significant differences (***p*** > 0.05) between these emotions. Another ***post-hoc*** Bonferroni adjusted pairwise comparison between each emotion of label-based method has also been conducted. The result shows no statistically significant differences (***p*** > 0.05) between happy and tensional, peaceful and sad. For other pairs, there are statistically significant differences (***p*** < 0.05). Combined with Figure 4, the results indicate that emotions with higher arousal like happy and tensional are more likely to be distinguished than emotions with low arousal like sad and peaceful for label-based method.

**Table 3:** A *post-hoc* Bonferroni adjusted pairwise comparison of each emotion pair between two methods.

**EmotionBox**	**Label-based method**	***p*** **-value**
Happy	Happy	0.606
Tensional	Tensional	**0.004**
Sad	Sad	0.240
Peaceful	Peaceful	**0.045**

***p***-value less than 0.05 means a statistically significant difference at a confidence level of 5***%*** and is presented in bold type.

To investigate the performance of generating different emotional music within each model, we also count the result of all the combinations between specific emotions at generating stage and emotions classified by subjects as shown in Table 4. From Table 4A, it shows that the arousal of music is more distinguishable than valence. For example, for the first row, 28% of happy samples were classified as tensional samples that have the same level of arousal but a different level of valence. However, a happy sample is rarely classified as a peaceful sample as they have a different level of arousal. This experimental result agrees with the observation that tempo is more determinant than the mode in forming happy-sad judgments as reported in Gagnon and Peretz (2003). In our work, the tempo and the mode are associated with arousal and valence of music, respectively. The classification of arousal and valence will be discussed in next section.

**Table 4:** The results of human classification for each combination between specific emotion at generating stage and emotion classified by subjects.

**(A)**				
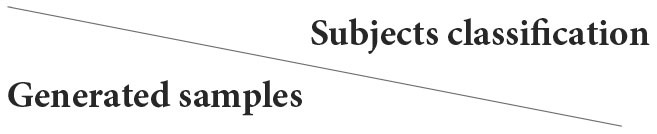	**Happy**	**Tensional**	**Sad**	**Peaceful**
Happy	71%	28%	0%	1%
Tensional	17%	74%	5%	4%
Sad	1%	8%	56%	35%
Peaceful	8%	4%	26%	63%
**(B)**				
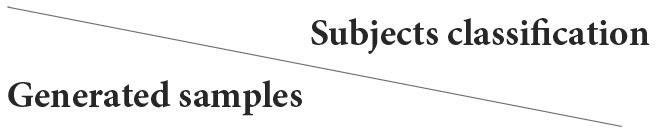	**Happy**	**Tensional**	**Sad**	**Peaceful**
Happy	74%	23%	0%	3%
Tensional	10%	90%	0%	0%
Sad	4%	18%	47%	31%
Peaceful	26%	28%	5%	41%

**(A)** The results of the EmotionBox. **(B)** The results of the emotion-label-based model.

From Table 4B, the classification accuracy is similar for high arousal music. However, for low arousal music, the classification accuracy in terms of both arousal and valence of emotion decreases significantly. For the last row, 26 and 28% peaceful samples were perceived as happy samples and tensional samples, respectively, which indicates that the label-based method has a poor performance on generating music with a low arousal emotion.

### Arousal and valence classification

**Figure 5:**
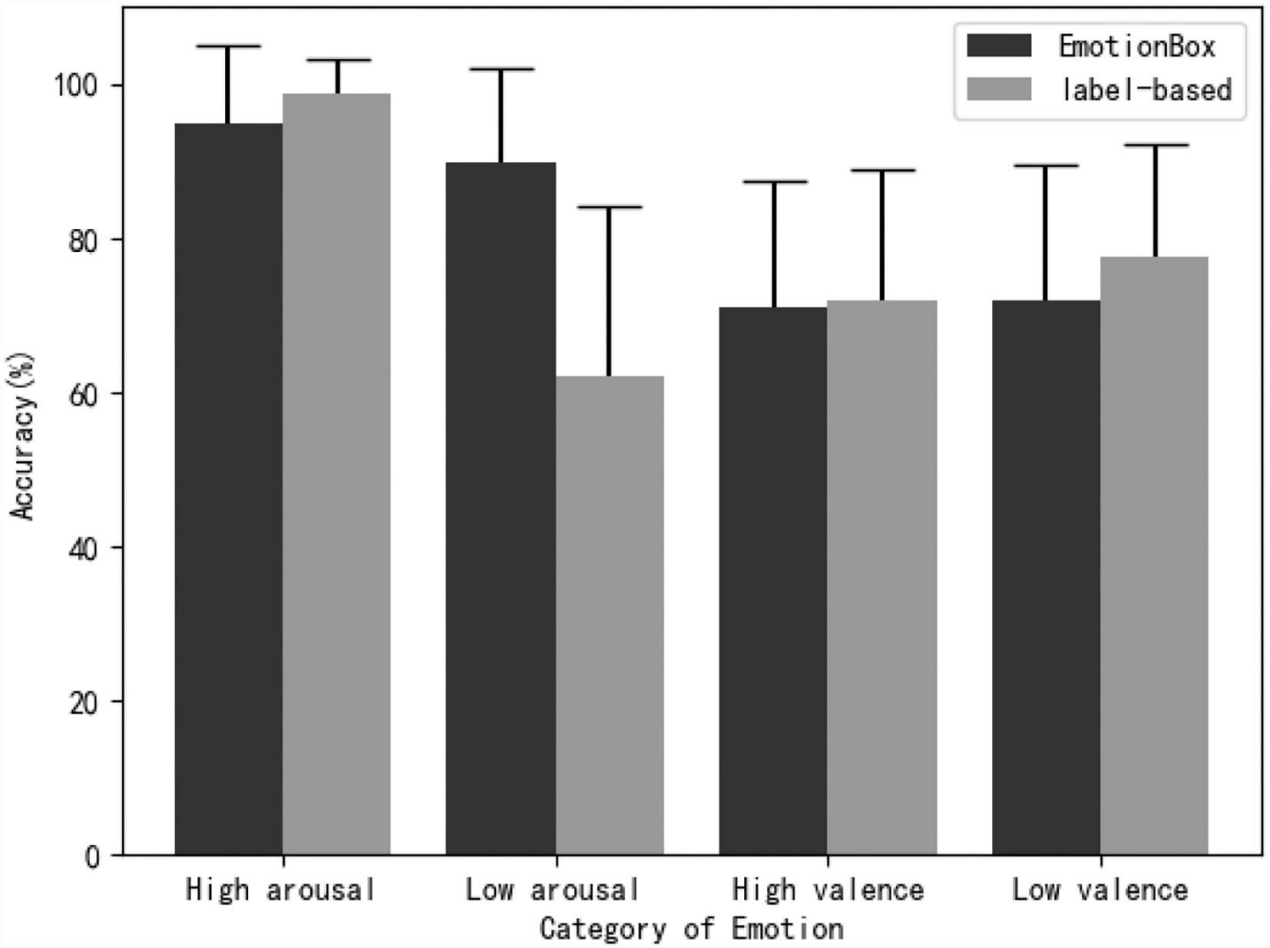
The mean accuracy and SD of subjective evaluation test for classifying generated music samples into arousal and valence categories.

Our proposed method uses note density and pitch histogram as features to present the arousal and valence of a specific emotion, respectively. To investigate whether these two features are suitable or not for training the deep neural networks, we calculated the accuracy of arousal and valence classification as shown in Figure 5. If the emotion specified during generating stage and the emotion classified by subjects have the same arousal or valence, the classification result will be calculated as correct. For example, if the emotion of a sample specified during generating stage is happy while classified as tensional by subjects, the classification result will be viewed as correct because of the same arousal of happy and tensional.

A two-way ANOVA is used with arousal and model set as within-subject factors to investigate how these two factors affect the accuracy of subjective experiments. The statistical results show that model [*F*_(1, 25)_ = 20.457, *p* < 0.01, partial η^2^ = 0.450] and arousal [*F*_(1, 25)_ = 42.989, *p* < 0.01, partial η^2^ = 0.632] have a significant effect on the accuracy of subjective experiments. The interaction of model and arousal has a significant effect on the accuracy of subjective experiments [*F*_(1, 25)_ = 43.846, *p* < 0.01, partial η^2^ = 0.637]. Another two-way ANOVA is also adopted with valence and model set as within-subject factors. The statistical results show that model [*F*_(1, 25)_ = 0.962, *p* = 0.346, partial η^2^ = 0.036] and valence [*F*_(1, 25)_ = 0.962, *p* = 0.259, partial η^2^ = 0.051] have no significant effect on the accuracy of subjective experiments. The interaction of model and valence shows no significant effect on the accuracy of subjective experiments [*F*_(1, 25)_ = 1.000, *p* = 0.327, partial η^2^ = 0.038]. Table 5 shows a *post-hoc* Bonferroni adjusted pairwise comparison between two methods in terms of arousal and valence.

**Table 5:** A *post-hoc* Bonferroni adjusted pairwise comparison of each arousal and valence conditions of the two methods.

**EmotionBox**	**Label-based method**	***p*-value**
High arousal	High arousal	0.325
Low arousal	Low arousal	<0.01
High valence	High valence	0.891
Low valence	Low valence	0.220

*p*-value less than 0.05 means a statistically significant difference at a confidence level of 5% and is presented in bold type.

It shows that the classification accuracy of EmotionBox is significantly higher than that of the label-based method on low arousal emotions. For other emotion categories, Table 5 shows that there is no significant difference between two methods for other three pairs. The tempo and the mode are relevant with note density and pitch histogram, respectively, in our work. Note density and pitch histogram further present arousal and valence, respectively. Without the limitation of note density, the label-based method tends to generate music with a faster tempo, which results in a low classification accuracy of the samples with low arousal emotions. This result means note density is a suitable feature to control the arousal of music.

### Limitations and outlook

However, there are still some limitations to the proposed method. First, the classification of valence is still challenging, which indicates that the valence of music cannot solely be presented by mode. A more appropriate presentation method of valence should be investigated in future work. Second, the generated music is more like an improvization. The model learns how to play the next note according to the previous notes whereas it has no idea about the structure of music. The structure of music is important and needs to be considered in the future work.

The EmotionBox can be used to help the composers create music with a specific emotion by providing various novel samples. By tuning the network's parameters, the EmotionBox can be a versatile assistant to create music. The combination of intelligent music composition and performance of music robot based on emotional computing is a promising approach for the future development of human-machine interaction, which provides a practical solution to eliminate the interaction barrier between humans and machines. Automatic emotional music may also be helpful for music therapy. Studies have shown neurological evidence that music effectively enhances auditory and language function through the human brain's plasticity (Hyde et al., 2009; Dittinger et al., 2017). Music therapies that utilize music as a treatment for tinnitus can leverage the plasticity in the auditory cortex and thus reduce the impact of tinnitus (Ellis et al., 2010). Some researchers have also shown that emotional music may support emotion recognition in children with ASD, and thus improve their social skills (Wagener et al., 2021). Music therapy often needs to avoid repetitive music. By tuning the networks parameters, the proposed method can generate non-repetitive music with a predefined emotion, which may be helpful for music therapy applications.

## Conclusion

In this work, we propose a music-element-driven automatic emotional music generator based on music psychology. This model does not need any music datasets with emotion labels that the previous methods required. The note density and the pitch histogram are chosen to present the arousal and valence of music, respectively. Then, different combinations of arousal and valence will be mapped to different emotions according to the Russell emotion model. Based on the specific note density and pitch histogram, our proposed method will be able to evoke listeners' different auditory perceptions and emotions. Subjective experimental results indicate that our proposed method has a significantly better performance in generating music with low arousal emotions. The results of the subjective listening test also indicate that note density is a suitable presentation for the arousal of music while more research studies should be carried out to find a more appropriate feature to convey the valence of music. The proposed method may have unique values for some music therapy applications.

## Data availability statement

The raw data supporting the conclusions of this article will be made available by the authors, without undue reservation.

## Ethics statement

The studies involving human participants were reviewed and approved by the Ethics Committee of the Institute of Acoustics Chinese Academy of Sciences. The patients/participants provided their written informed consent to participate in this study.

## Author contributions

KZ: writing. RM, KZ, and JS: methodology. CZ and XL: supervision and editing. JS: writing-review. JC: database. JW: evaluation. XW: data analysis and modification. All authors contributed to the article and approved the submitted version.

## Funding

This work was supported by the National Science Fund of China (Grant Nos. 12074403 and 11974086), the Open Research Project of the State Key Laboratory of Media Convergence and Communication, Communication University of China, China (Nos. SKLMCC2021KF014 and SKLMCC2020KF005). This work was supported by National Key Research and Development Project (2021YFB3201702).

## Conflict of interest

The authors declare that the research was conducted in the absence of any commercial or financial relationships that could be construed as a potential conflict of interest.

## Publisher's note

All claims expressed in this article are solely those of the authors and do not necessarily represent those of their affiliated organizations, or those of the publisher, the editors and the reviewers. Any product that may be evaluated in this article, or claim that may be made by its manufacturer, is not guaranteed or endorsed by the publisher.

## Footnotes

^1^The codes are available on https://github.com/KaitongZheng/EmotionBox.

^2^The training data can be found on http://www.piano-midi.de/.

^3^The subjective listening test files can be found on https://github.com/KaitongZheng/EmotionBoxDEMO.
